# Dialysis Patients With Restless Leg Syndrome: Can We Relieve Their Suffering?

**DOI:** 10.7759/cureus.10053

**Published:** 2020-08-26

**Authors:** Marina Salib, Areeba N Memon, Asavari S Gowda, Bhavana Rallabhandi, Erjola Bidika, Hafsa Fayyaz, Ivan Cancarevic

**Affiliations:** 1 Internal Medicine, California Institute of Behavioral Neurosciences & Psychology, Fairfield, USA; 2 Research, California Institute of Behavioral Neurosciences & Psychology, Fairfield, USA; 3 Neurology, California Institute of Behavioral Neurosciences & Psychology, Fairfield, USA

**Keywords:** rls, esrd, ckd, hemodialysis

## Abstract

Restless leg syndrome (RLS), also called Willis Ekbom disease, can be described as an unpleasant feeling that intensely urges the patients to move their lower limbs. RLS is classified into primary and secondary. It is one of the common complications in hemodialysis patients, and it impairs patients’ quality of life. Unfortunately, it is an underdiagnosed and undertreated disorder. In this review article, we performed a literature search using the PubMed database to compare different treatment modalities for RLS in patients with end-stage renal disease (ESRD) on regular hemodialysis. Many of the non-pharmacologic modalities of treatment are cost-effective and safer than pharmacologic therapy. Given the small sample size of the studies and short follow up duration, we should consider conducting studies on a larger number of patients and for longer periods of time to assess the efficacy and safety of different treatment patterns for RLS in hemodialysis patients. We hope to raise awareness about this neurologic condition in hemodialysis patients.

## Introduction and background

A 78-year-old male patient has end-stage renal disease (ESRD) on regular hemodialysis presented to the emergency room (ER) with gangrene in his left lower limb. While interviewing the patient, the physician noticed that patient was scratching his skin vigorously. He was angry, complained about not being able to sleep all night, and that he had sudden, involuntary movement in his lower limb that was more frequent at rest. The patient felt depressed and was not interested in his life anymore. He was asking to stop his hemodialysis sessions and to receive palliative care [1].

Chronic kidney disease (CKD) patients treated with different dialysis modalities may suffer from many mental and physical complications. They may have complications of their original kidney pathology, adverse effects of their medications, and complications of dialysis therapy. Restless leg syndrome (RLS) is one of these common complications in hemodialysis patients. The prevalence of this sensorimotor neurologic condition in hemodialysis patients has been estimated between 6.6% and 80% compared to 7%-24.1% in the non-dialysis population [2].

According to the International Restless Leg Syndrome Study Group (IRLSSG), RLS is an uncomfortable sensation that urges the patient to move his/her lower limb, as moving helps alleviate this feeling [1]. It may occur in other body parts, but it is more common in the lower limbs [3,4]. Sometimes the patient may describe it as feeling that something is crawling on his/her skin or bone pain in his/her lower limb [4]. It can be associated with periodic limb movements (PLM) in the evening, as this condition is exacerbated by relaxation [5-7]. RLS is classified into primary and secondary. Primary RLS can be due to genetic factors since 40% of patients have a positive family history [8]. Secondary RLS is associated with iron deficiency, diabetes mellitus, multiple sclerosis, peripheral neuropathy, rheumatoid arthritis, and renal failure [9]. Some of the medications known to elicit RLS symptoms are antihypertensives, histamine receptor blockers, and antidepressants [10].

RLS pathophysiology is not entirely understood; it can be due to dopamine imbalance, decreased iron stores in the brain, or iron regulation disturbances [11]. Smoking, caffeine intake, alcohol intake, hypertension, diabetes mellitus, and female sex are risk factors associated with RLS [12].

RLS impairs patients’ quality of life since it has negative effects on patients’ mental and physical health. It is associated with impaired sleep quality, increasing daytime sleepiness, and increased risk of using sedative-hypnotics [12]. There is a positive correlation between fatigue severity and RLS severity [12]. RLS is also associated with depression, anxiety, and sexual dysfunction [12,13]. It is associated with a significantly higher risk of cardiovascular morbidity and mortality [12]. According to previous studies, RLS symptoms in the uremic patient are more severe than in patients with primary RLS [14].

Some studies have been done to estimate the associated risk factors, pathophysiology, and treatment options. There are no specific laboratory or imaging studies that can be used to confirm RLS diagnosis. The diagnosis depends only on IRLSSG diagnostic criteria, so diagnosis is purely clinical [11]. Although it is a common disorder, it is neglected in dialysis centers and frequently not accurately diagnosed due to overlap with other sensory and motor conditions in the lower extremities [15]. Unfortunately, it is an underdiagnosed and undertreated neurologic disorder.

The high prevalence of RLS in dialysis patients and its negative effect on the patients’ quality of life should raise more concern about treatment options for this disease [16]. Exploring and providing more treatment options for RLS, including pharmacologic and non-pharmacologic methods, is required. Non-pharmacologic therapies such as intradialytic stretching exercise and shifting the patient to different dialysis modalities are safer than pharmacologic treatment, as dialysis-patients have other comorbidities and impaired elimination of different medications [17-19]. Searching for safe, effective, low-cost treatment for RLS can help to decrease dialysis patient complaints and improve their quality of life.

In this review article, we collect information from previously published articles and studies using the PubMed database to find out different treatment options for RLS in hemodialysis patients. We hope to raise awareness about the prevalence, diagnosis, and treatment of this condition in dialysis patients.

## Review

Prevalence and risk factors

Many studies have been conducted to estimate the prevalence of and risk factors associated with RLS in CKD patients on regular hemodialysis. The prevalence and associated risk factors with RLS in hemodialysis patients are highly variable in different populations. Based on the study conducted on 221 patients by Turk et al., the prevalence of RLS in hemodialysis patients in Turkey was estimated to be 16.8% [12]. The same study proclaimed that RLS is more prevalent in females (odds ratio [OR] = 0.435; 95% CI = 0.206-0.918; P = 0.029) [12]. Also, high serum ferritin (OR =1.002; 95% CI =1.000-1.003; P =0.009) and c-reactive protein (CRP) (OR = 1.021; 95% CI = 1.021-1.039; P = 0.02) were found with other risk factors for RLS in these patients [12]. In another study by Saraji et al. involving 260 hemodialysis patients from Iran, the prevalence of RLS was 55% [20]. Higher mean age (P = 0.002), and longer dialysis duration (P = 0.008) were the associated risk factors with RLS, but this study didn’t find any association between gender and RLS [20]. Lin et al. reported that RLS prevalence was 20.44% in the Chinese population, and it was more common in females (OR = 2.729, P = 0.032), and alcoholic patients (OR = 4.716, P = 0.022) [15]. A case-control study in Taiwan by Tsai et al. reported that longer duration on regular hemodialysis therapy (OR = 2.32; 95% CI = 1.23-4.39; P = 0.002), and lower levels of high-density lipoprotein (HDL) cholesterol (<40 mg/dL in men; <50 mg/dL in women) (OR = 2.73; 95% CI = 1.44-5.15; P = 0.009) are significantly associated with RLS in hemodialysis patients [4]. Another cross-sectional study in Saudi by Wali and Alkhouli found that RLS prevalence in hemodialysis patients was 19.4%, and it was more prevalent in patients with increased BMI (P = 0.001), and in patients taking anticoagulants (P = 0.035), or aspirin (P = 0.037) [21].

Data of the selected studies are documented in Table 1.

**Table 1:** Prevalence and risk factors for RLS RLS, restless leg syndrome; CRP, c-reactive protein; BMI, body mass index; HDL-C, high-density lipoprotein cholesterol

Author and Year of Study	Country	Number of Participants	Prevalence	Risk Factors
Turk et al., 2018 [12]	Turkey	221	16.8%	Female sex, high serum ferritin and high CRP
Saraji et al., 2016 [20]	Iran	260	55%	Higher mean age, and longer dialysis duration
Tsai et al., 2019 [4]	Taiwan	467	12.6%	Longer dialysis duration and low HDL-C level
Lin et al., 2019 [15]	China	137	20.44%	Female sex and alcoholics
Wali and Alkhouli, 2015 [21]	Saudi	355	19.4%	Increased BMI and patients taking aspirin and anticoagulants

The previous studies showed that the prevalence of RLS in hemodialysis patients is highly variable in different populations. It was 16.8% in Turkey, 55% in Iran, 20.44% in China, and 19.4% in Saudi [12,15,20,21]. Global variation in the prevalence of RLS can be attributed to some demographic differences, racial factors, and using different diagnostic criteria for RLS [22]. We can also consider different study methods. Multiple risk factors are associated with RLS in the dialysis population. Most studies reported female sex, alcohol, increasing BMI, higher serum ferritin level, and CRP as risk factors associated with RLS. Some medications, such as aspirin and anticoagulants, are associated with RLS development in dialysis patients. By looking at these risk factors, we can conclude that RLS is more prevalent in hemodialysis patients as they have chronic inflammatory status with high serum ferritin and CRP levels.

Pathophysiology

The pathophysiology of both idiopathic and secondary RLS is still a subject of discussion. A limited number of studies have been conducted to find out the pathophysiology of RLS in uremic patients. Kavanagh et al. proclaimed that in addition to the role of dopamine and iron disturbance in the pathophysiology of RLS, hyperphosphatemia, anemia, and other psychological factors play an essential role in RLS pathophysiology in uremic patients [23]. Einollahi et al. explained that as dopamine recycling is an intracellular enzymatic reaction that requires iron as a cofactor, deficient iron stores in the central nervous system (CNS) can be a cause of secondary RLS [11]. This theory can explain secondary RLS occurrence in anemic hemodialysis patients. The results were supported by brain imaging and autopsy findings that showed deficient iron stores in these patients’ brains [11]. Higuchi et al. found that RLS can be associated with chronic inflammation and oxidative stress in uremic patients [24]. The study based this conclusion on previous studies showing that RLS was more prevalent in patients with higher CRP, higher serum ferritin, and lower transferrin saturation [24]. Takaki et al. reported that RLS in uremic patients can be related to iron deficiency and high phosphorus levels [13].

The studies that are conducted to explain the pathophysiology of RLS in uremic patients are very limited. The suggested mechanisms for RLS pathophysiology considered that imbalance of iron and dopamine in the CNS can be the leading cause of RLS. They proposed that the chronic inflammatory status and the oxidative stress in hemodialysis patients can be significant contributing factors in RLS pathophysiology. Low serum iron and high serum phosphorus levels are also believed to play a role.

Complications of RLS and its effect on patient’s life

RLS has negative impacts on patients’ quality of life in multiple different ways. RLS’s common complications are poor sleep quality, fatigue, depression, and periodic limb movement of sleep (PLMS). Rohani et al. reported that RLS is associated with insomnia, daytime sleepiness, shorter sleep duration, and using sedative-hypnotic medications [25]. Giannaki et al. reported that fatigue is a prevalent symptom in chronic renal failure (CRF) patients, but it is more prominent in patients with RLS [26]. Fatigue can be related to impaired sleeping quality and decreased sleeping time in these patients [26]. Another study by Giannaki et al. found that hemodialysis patients with RLS showed atrophy in the thigh muscle compared to patients without RLS [18]. Bambini et al. reported that PLMS is more prevalent in ESRD patients with RLS (P = 0.003) [27]. Takaki et al. reported that RLS is significantly associated with higher anxiety prevalence in hemodialysis patients [13]. On the other hand, Sakkas et al. found that although RLS is associated with other morbidities, it does not affect the mortality rate in ESRD patients and still the cardiovascular cause is the first contributor to the mortality rate in ESRD [28]. The same results were reported in a study by Baiardi et al. conducted on 128 ESRD patients on hemodialysis [29]. The study concluded that RLS doesn’t affect the mortality rate in hemodialysis patients [29].

The aforementioned studies concluded that RLS significantly decreased the quality of life in hemodialysis patients. It is associated with poor sleep, anxiety, PLMS, sedative-hypnotic medications usage, and fatigue. Although RLS is a very distressful condition, it is not associated with higher mortality in hemodialysis patients. Treating this disease may help to alleviate dialysis patients’ suffering and improve their life quality.

Treatment of RLS

Many treatment options are available for RLS, both pharmacologic and non-pharmacologic. While choosing the appropriate treatment for each patient, we should consider his/her general condition, financial status, and other comorbidities and non-kidney conditions.

Pharmacologic Treatment

Since dopamine dysfunction and iron deficiency in the CNS are among the most commonly used mechanisms to explain RLS pathophysiology, Ekbom et al. suggested using dopamine agonists, levodopa, and iron supplements for pharmacological treatment of RLS [30]. He also mentioned opioids and benzodiazepines as other options for treatment [30]. Another study by Garcia-Borreguero et al. recommended using non-ergot dopamine agonists as first-line treatment for RLS but it found that augmentation of the symptoms after using these drugs is the main side effect in some patients [31]. Pellecchia et al. conducted a clinical trial on 11 hemodialysis patients to compare the efficacy of different dopaminergic drugs [32]. The study assessed the severity of RLS symptoms using the IRLSSG questionnaire after giving the patients either ropinirole or levodopa, the results showed that ropinirole is superior to levodopa in improving RLS symptoms [32]. Another clinical trial was conducted to evaluate gabapentin as a treatment for RLS in dialysis patients [33]. A double-blind, placebo-crossover clinical trial was conducted by Thorp et al. on 16 hemodialysis patients [33]. It showed significant improvement in RLS symptoms (P < 0.001) after using gabapentin for six weeks, compared with patients who used a placebo [33]. Finally, there were no studies to assess using opioids for RLS treatment in dialysis patients [34].

Non-Pharmacologic Treatment

A study conducted by Santos et al. to evaluate the effect of parathyroidectomy on dialysis patients [3]. The study was conducted on 10 dialysis patients with hyperthyroidism, hyperphosphatemia, and diagnosed with RLS. After parathyroidectomy, the patients showed significant improvement in RLS severity [3]. Rad et al. reported that using cool dialysate for hemodialysis can help alleviate RLS symptoms by enhancing toxins removal from the blood and decreasing creatinine and urea levels [35]. A study conducted by Shahgholian et al. compared the effect of reflexology, stretching exercise, and placebo on RLS severity index in dialysis patients showed significant improvement in RLS severity with reflexology and stretching exercise compared to placebo [17]. Song et al. conducted a metanalysis on the effect of exercise on RLS, fatigue, depression and sleeping quality in hemodialysis patients, and he reported that exercise can be effective in improving symptoms of RLS (P < 0.001), fatigue (P < 0.001) and depression (P < 0.001) [36]. Mohammadi et al. performed a single-blind, randomized controlled trial, and he found that near-infrared light can decrease symptoms of RLS in hemodialysis patients [37]. Kahvecioglu et al. declared that there is an improvement of RLS in hemodialysis patients after renal transplantation and normalization of the kidney functions [38]. In a clinical trial conducted by Nasiri et al. to assess the effect of massage with olive oil after dialysis sessions on RLS patients, the study stated that olive oil can be used as a complementary treatment for RLS since it improves the symptoms significantly [39]. Another study by Hashemi et al. reported improvement with the use of lavender oil for massage [40]. Hosseini et al. studied the effect of vibration on RLS severity and reported that vibration improved RLS symptoms in hemodialysis patients [41].

Given the small sample size of the studies, short follow up duration, and the lack of larger randomized controlled trials of the different treatment options, we should consider conducting more studies with more participants to assess the efficacy and safety of different treatment modalities for RLS in hemodialysis patients. But in general, many of the non-pharmacologic modalities of treatment are cost-effective and safer than pharmacologic therapy.

## Conclusions

Diagnosing and treating RLS in CRF patients on regular dialysis can be challenging. RLS diagnosis depends on the medical awareness of this condition. There are two categories of RLS treatment in this population, pharmacologic and non-pharmacologic. Pharmacologic treatment involves dopamine agonists, gabapentin and, iron supplements, while non-pharmacologic treatments involve using massage, reflexology, and cool dialysate to alleviate this condition. We should encourage conducting more studies on a larger number of patients, for a longer duration of follow up to find out the appropriate, safe, cost-effective treatment for this condition in dialysis patients.
